# Quality of Informed Consent and Interface Usability in Primary Care e-Consultation: Cross-Sectional Study

**DOI:** 10.2196/78483

**Published:** 2026-02-09

**Authors:** Caitlin Parfitt-Ford, Lisa Ballard, Adriane Chapman

**Affiliations:** 1 School of Electronics and Computer Science Faculty of Engineering and Physical Sciences University of Southampton Southampton United Kingdom; 2 Primary Care, Population Science, and Medical Education Faculty of Medicine University of Southampton Southampton, England United Kingdom; 3 Southampton Biomedical Research Centre National Institute for Health Research Southampton, England United Kingdom

**Keywords:** remote consultation, informed consent, electronic consultation, telemedicine, digital triage, privacy, ethics, medical informatics

## Abstract

**Background:**

Patient autonomy through informed consent is a foundational ethical principle for health care practitioners. Online consent processes risk producing “consent in name only,” using manipulative or confusing user interfaces to extract consent artificially. This presents a significant danger for safe and ethical remote consultations for primary care providers, which often extract significant amounts of sensitive personal data.

**Objective:**

This study aims to examine the quality of consent obtained through both currently used and novel consent acquisition interfaces for remote e-consultations between a patient and a primary care provider.

**Methods:**

A total of 55 adult participants in the United Kingdom completed an interaction with a mock-up e-consultation system’s consent interface for data processing, with 54 completing the full study protocol. The participants were then asked questions regarding what they had provided consent for and the usability of the interface. These responses led to the calculation of an industry-standard System Usability Scale (SUS) score and a novel Quality of Informed Consent Collected Digitally (QuICCDig) score.

**Results:**

Users perceiving interfaces to be more usable (with a greater SUS score) were statistically significantly (n=54; *P*=.004) correlated with an increase in the quality of consent collected from those users (with a higher QuICCDig score). Nonetheless, both existing and novel user interfaces for collecting e-consultation consent were rated poorly, achieving a maximum SUS letter grade of “F.” In total, 45% (25/55) of all the participants reported not recalling making a privacy-related decision at all during their consultation, and 87% (48/55) did not recall being offered any alternatives to e-consultation.

**Conclusions:**

The findings demonstrate that current methods for collecting consent in telemedical applications may not be fit for purpose and potentially fail to collect valid informed consent. However, increased usability scores from users do appear to drive improvements in the quality of consent collected. Therefore, decision-makers should place importance on high-quality interface design when building or procuring these systems. We have also provided the QuICCDig score for further use.

## Introduction

Remote electronic systems for consultations between patients and their primary care providers have been growing rapidly in use since they were first conceptualized—growth that was accelerated by the pressures of the COVID-19 pandemic on the health care system [1]. These systems allow patients to obtain medical advice without having to physically travel to a clinic or speak to a primary care provider over the phone by answering questions and uploading images to an online system for a health care professional to review, thereby expanding access to health care. They allow for rapid symptom triage, which may increase primary care operational efficiency [2]; among some patient populations, they may also lead to increased honesty with health care providers when consulting about symptoms perceived to be embarrassing [3].

Adoption of these systems has varied across international primary care environments. Around the world, asynchronous primary care “e-consultations” conducted via online platforms are known to be used in Norway, Spain, Sweden, the United Kingdom, and the United States [4,5]. In the United Kingdom, access to online consultations from patients to their general practitioner (GP) has been compulsory for GP practices since the April 2021 financial year’s contract [6], as part of the National Health Service (NHS) Long Term Plan from 2019 [7]. However, partly owing to unforeseen pressures of the COVID-19 pandemic to implement the technology in a crisis-driven fashion, the success of the rollout has been variable, with some practices seeing substantial efficiency improvements, while others have since reverted to a “strategically traditional” model for meeting the needs of their patient population [8].

Ethical challenges relating to informed consent in telemedicine are nothing new—home monitoring systems have faced them for some time [9]. However, despite the increased uptake of e-consultations [10], issues of safety and consent have not been well evaluated in existing literature [4]. This lack of focus on safety, combined with technocentric policymaking, risks leading clinicians and technologists alike to be overeager to implement new technologies without adequate consideration of the implications for patients’ bioethical and legal rights [11]. Regulators and lawmakers internationally have been slow to respond to advances in telemedicine, muddying the waters further [4]—even as disparities in patient understanding of digital health information across age groups and health numeracy have been demonstrated [12].

In the development of websites more broadly, impetus for collecting consent from users for data processing largely comes from regulatory requirements rather than ethical frameworks. Standards for ethical practice for software engineers have been described as “toothless” and criticized for “ethics-washing” without actually changing corporate incentives [13]. Perhaps unsurprisingly, many examples exist of users’ data being collected on a “legal minimum” basis or even without proper consent at all [6,7]. Bollinger et al [14] found that, of approximately 30,000 websites studied, 94.7% contained potential European Union General Data Protection Regulation violations related to cookie consent, although that figure is based on automated detection without human verification. In some cases, interfaces are deliberately designed so that users do not remember their choices, even when they regret them upon being reminded of them [9]; however, this has become such standard practice that users simply consider it part and parcel of their internet experience [15].

This resigned acceptance of poor ethical standards in the context of the internet presents a challenge for facilitating ethical telemedical interactions, as the ethical and legal standards for consent in medical contexts are substantially more stringent. Notably, for example, guidance in the United Kingdom consistently highlights the need for clinicians to obtain informed consent from patients for examination and assessment and not just subsequent treatment [16-19]; indeed, government guidance makes it clear that even implied consent for measuring blood pressure by a patient holding out an arm may require the patient “receiving appropriate information” first [20]. The courts have confirmed this; in *R v Hallstrom*, Mr Justice McCullough writes “it goes without saying that, unless clear statutory authority to the contrary exists, no one is […] even to submit himself to a medical examination without his consent” [21]. Failure to collect consent appropriately may be considered a reportable adverse event [22].

There are no currently accepted systems that evaluate clinical informed consent collected through entirely digital systems with no human-in-the-loop involvement. Usability, defined by International Organization for Standardization 9241-11:2018 as the “extent to which a system, product or service can be used by specified users to achieve specified goals with effectiveness, efficiency and satisfaction in a specified context of use” [23], is measured using well-established metrics such as the Post-Study System Usability Questionnaire or the System Usability Scale (SUS) [24,25]. However, these metrics, when used alone, could, for instance, result in high scores for interfaces that are extremely user-friendly but fail to convey important information, as patients’ feelings of being informed in the context of clinical consent may not actually relate to objective measures of knowledge [26]. Therefore, a clear gap exists for a metric to assess the quality of digitally collected informed consent.

This work analyzed the quality of consent that can be collected through remote patient–primary care provider e-consultations. To do this, we (1) developed a scoring system by which consent gathered for electronic patient–primary care provider medical consultations can reliably be evaluated, (2) established an understanding of the validity of current strategies for consent acquisition in remote patient–primary care provider e-consultation via the internet, and (3) evaluated whether changes in user interface design could impact the quality of consent collected.

## Methods

### Overview

This research developed a novel questionnaire and scoring system, Quality of Informed Consent Collected Digitally (QuICCDig), for evaluating the quality of health care informed consent collected digitally, based on a synthesis of existing systems from health and technology consent research. A selection of real online consultation systems was then evaluated, using both QuICCDig to evaluate the quality of the consent collected and the industry-standard SUS to evaluate the perceived usability of the interface.

### QuICCDig Development

In this study, we synthesized QuICCDig from an analysis of multiple existing criteria for consent evaluation across both health care and digital technology. A narrative literature review was conducted, investigating current methods used for evaluating the quality of consent collection in medical consultations and digital interactions. PubMed, Google Scholar, and HeinOnline were searched for terms including “quality of informed consent,” “digital consent evaluation,” “medical consent evaluation,” “digital consent quality,” and “medical consent quality” without restricting to a particular date range, location of publication, or format of publication.

Search results were then assessed for relevance. In particular, results with no full text available to the researchers were excluded as were results whose full text did not contain evaluation methodologies for consent quality or whose methodology was based on parameters such as refusal rate rather than the quality of the consent process itself. Methodologies that were specific to the consent process for a particular subspeciality were excluded, but methodologies used for the evaluation of consent for clinical research were included, as it was hypothesized these might relate to more relevant data-sharing exercises. Where results were already referenced in a broader piece of work, such as a systematic review, the original work was excluded in favor of the broader work to simplify identification of themes.

### Interface Evaluation Study Design

To understand how well existing digital consultation systems manage the challenge of acquiring informed consent, we (1) reviewed existing consultation systems approved for use in the United Kingdom to evaluate what consent interfaces they use, (2) created a series of interactive mock-up interfaces (defined as midfidelity interactive prototype user experiences that allow users to complete a specific interaction as relevant for the study) for consent collection in the context of a digital consultation, and (3) provided participants with the interface and subsequently administered the SUS and QuICCDig questionnaires to evaluate the usability of the interface and the quality of the consent collected through it.

All 9 online consultation systems approved for use in the United Kingdom NHS Digital Buying Catalogue [27] as of January 2023 that supported “self-help and signposting” or “symptom checking” were reviewed, of which 7 were noted to be suitable for primary care patient-provider remote consultation use. Only 2 contemporary practices for consent were identified in these 7 systems: either “link and checkbox,” in which users are shown a checkbox to confirm their acceptance of privacy information or “summary and checkbox,” in which users are shown a checkbox to confirm their acceptance along with a brief explanation of what they are providing consent for and a link to read more. The design of these interfaces reflects different modalities of user interaction as well as different amounts of content presented initially to the user; these differences may reflect attempts to promote user recognition of familiar interaction paths within known user interface paradigms while also allowing appropriate use of users’ background knowledge, mapping to the Nielsen design heuristics of “recognition rather than recall” and “match between system and real world” [28].

On the basis of these real consultation systems, 9 mock-up user interfaces were designed, with 2 based on contemporary “link and checkbox” or “summary and checkbox” consent practices and the remainder being novel interfaces not seen to be used in existing approved software (Table 1; for screenshots, refer to Multimedia Appendix 1). Checkbox, drag-and-drop, and swipe interfaces were investigated, as previous research has shown that the quality of consent collected and the perceived usability of the interface may vary across these interface types [29,30].

Participants’ demographic data were collected, and they were then presented with a brief to complete a simulated consultation related to a low-acuity mental health presentation. This presentation was chosen because it may result in greater concerns about privacy and confidentiality than a physical health presentation [31,32]. Participants were randomized by computer to interact with 1 of the 9 interfaces, and metadata regarding interactions, such as click heat maps and interaction times, were recorded by the system. The only differences between the interfaces were their consent collection techniques—the other elements were identical. Each participant was only shown the single interface to which they were randomly allocated.

After completing the interaction, participants were presented with a survey evaluating their impressions. First, participants were asked to complete the standard “SUS” questions [24]; they were then presented with the QuICCDig questionnaire described earlier.

**Table 1:** Mock-up interface reference table according to their features.

	Blanket statement with link to privacy policy	Summary of privacy policy and details page	All policy information contained on a single page
Checkbox	Interface 1	Interface 4	Interface 7
Drag and drop	Interface 2	Interface 5	Interface 8
Swipe	Interface 3	Interface 6	Interface 9

### Ethical Considerations

This research was approved by the University of Southampton Faculty of Engineering and Physical Sciences Ethics Committee (FEPS/ERGO/78443). Participants were presented with signposting information for support if the study caused any discomfort or distress and informed of the voluntary, anonymous nature of the study. Informed consent was gained from all participants before their participation, and participants were reminded of their ability to opt-out at any time. Participants were not compensated for their participation. No personal data were used in the analysis.

### Recruitment and Statistical Power

Participants aged 18 years or older who had previously interacted with a GP were recruited via snowball sampling from an initial set of posts on social media as well as from advertisements placed on a university campus. A target of 113 participants was set based on a planned linear multiple regression analysis (with an anticipated f^2^ value of 0.15, an α value of .05, and a power of 0.8). In actuality, 93 participants were recruited; 66 (71%) completed their interactions enough to answer at least the SUS questions, and 55 (59%) completed the survey well enough to calculate a QuICCDig score. Of these 55 participants, 45% (25/55) had formal computer science-related education, 27% (15/55) had formal medical education, and 76% (42/55) had been prior users of e-consultation technology.

**Figure 1:**
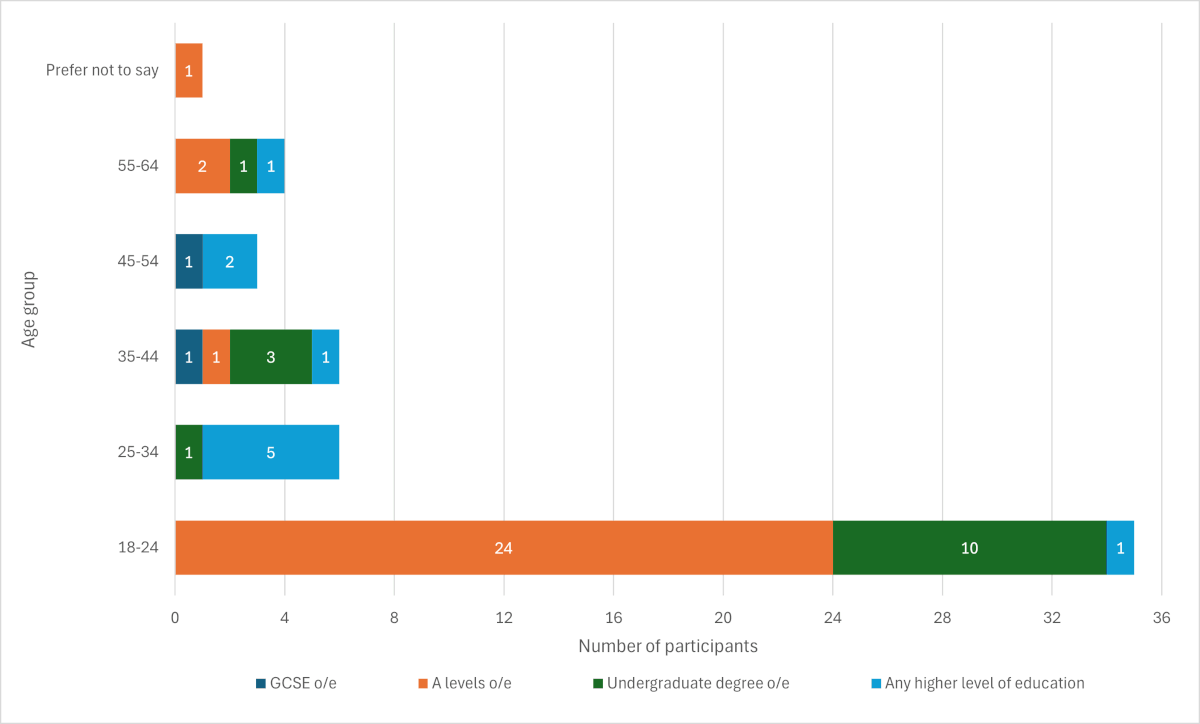
Demographic breakdowns of participants. GCSE: General Certificate of Secondary Education; o/e: or equivalent.

In total, 1 (1%) participant who completed the QuICCDig questionnaire could not have their SUS score calculated due to a technical issue, leaving 54 (58%) of the recruited participants with valid scores for both SUS and QuICCDig. Demographic breakdowns of participants are included in Figure 1.

## Results

### QuICCDig Synthesis

A total of 11 key papers were identified, and their content was then reviewed for themes. After excluding themes that were either impossible to measure reliably without a human in the loop and were thus impractical to include in an online primary care patient-provider e-consultation system, such as the use of repeat-back testing [33,34], or items that could automatically be collected in a digital system without having to ask a patient (and therefore did not need to be explicitly asked), such as time to complete a consent process or parts of a website that were consulted, 14 key themes were identified as being used in current evaluation criteria. From these themes, corresponding questions were synthesized for inclusion in the QuICCDig metric. The themes included and the questions produced are presented in Table 2, while themes identified but excluded are detailed in Table 3. The scoring metric for these questions is detailed in Multimedia Appendix 2.

**Table 2:** Quality of Informed Consent Collected Digitally (QuICCDig) themes and questions produced.

Theme	Study	Question
Ability to complete the task	[29,35]	“Were you able to successfully complete your e-consultation?”
Perceived ease of use	[14,15]	“How easy did you find it to complete your e-consultation?”
Perceived quality of description and explanation of processes and how they work	[34-37]	To be asked before the main body of the study, alongside the demographic questions “What do you think happens during an e-consultation, and where does the information go?” Asked with the rest of the QuICCDig questions after the main body “Did you get an explanation of what an e-consultation involves?” “How well do you feel you understand e-consultations now?” “Did your understanding change from what you thought happened in an e-consultation before? If so, what changed?”
Objective understanding of processes and how they work	[35,38]	“How long will your consultation data be stored?” “Which of the below might have access to some data collected during the consultation? [Select multiple: Me, my healthcare provider, other healthcare providers, the company that runs the consultation software, Google, Facebook].”
Why it is being done and the benefits to the patient	[35-38]	For each group identified in the question about who will have access to consultation data “Why will NAME have access to your data?”
Risks to the patient inherent to the procedure	[34,36-38]	“Which of the below are risks involved in the e-consultation process?”
Alternatives	[34,36-38]	“Do you remember being told about the alternatives to using an e-consultation?” If yes, “What alternatives were there?”
Recollections of consent	[34,35]	“Do you remember making any privacy-related decisions during your e-consultation?” If yes, “What do you remember deciding?”
Effect on privacy and confidentiality	[37]	“What impact will this e-consultation have on the confidentiality of your medical records?”
Degree of satisfaction with the decision-making and consent process	[34,37,39]	“How satisfied with the decision-making process for whether you wanted to continue with an e-consultation were you?”
Whether the patient knew who to ask questions to	[34,37]	“If you had questions about how your e-consultation would work, or how your data would be used, who would you ask?”
How to revoke consent	[35]	“If you wanted to change your mind about your e-consultation data being used, what would you do?”
Patient’s feeling of involvement with the process	[34,35,39]	“To what extent did you feel informed and involved with the process of consenting for your e-consultation? [Select one: Not at all, Somewhat, Enough, More than enough, Too much]”
Anything the patient felt was missing	[35]	“Was there anything else you would have liked to have known that you weren’t told about?”

**Table 3:** Themes identified in the literature but not included in the Quality of Informed Consent Collected Digitally (QuICCDig) scoring system.

Theme	Study	Justification for lack of inclusion
Whether the patient was asked for consent	[34]	In the context of an interaction that is guaranteed to present a prominent consent screen, asking users whether they were prompted for consent would not be expected to yield useful data compared to an in-person medical interaction, where it could be forgotten or made nonobvious.
Timing of information being given	[34,36]	Specific times at which consent information is presented can be automatically collected from an online system but are also less likely to be relevant in the context of an asynchronous patient–primary care provider interaction.
Who gave the information to the patient	[34]	Consent information in online systems, such as primary care patient-provider e-consultation systems, is consistently provided through an automated online interface.
Whether the patient was asked to repeat the explanation	[34]	Assessing repeated explanations for coherence and relevance would be essentially impossible to achieve without the involvement of a clinician, which is not practical for the purposes of a patient-provider e-consultation in primary care.
Questions relating to research consent	[37]	The scope of the QuICCDig score is limited to clinical consent for consultation and assessment.
Questions relating to ignoring consent dialogues and continuing use of the application in any event	[35]	In all the interfaces used and identified, it is not possible for the user to proceed with the consultation without interacting with the consent interface.
Questions relating to specific fine-grained control of (eg, cookies)	[35]	Control of specific aspects of the technical function of the system is outside the scope of the QuICCDig score; rather, the overall consent process is evaluated.
Questions about which parts of the website were consulted	[35]	In this study and in most similar interfaces identified in real-world use in the National Health Service Digital Buying Catalogue, interfaces are presented as single-page applications rather than multipage websites with multiple interaction paths.
Time to complete the consent and consultation process	[29]	This can be captured automatically by online systems.

### Interface Evaluation Study

**Figure 2:**
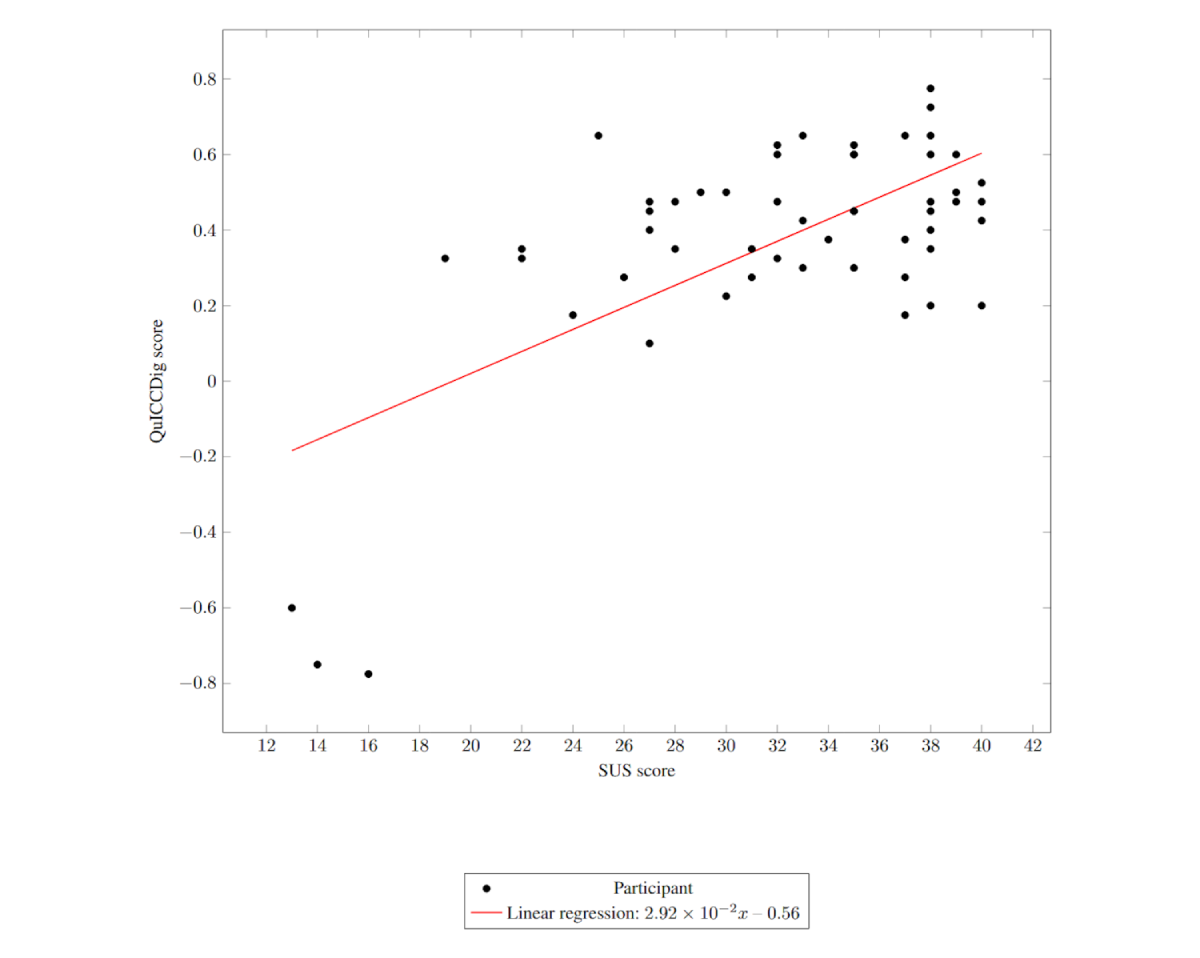
Quality of Informed Consent Collected Digitally (QuICCDig) score against System Usability Scale (SUS) score.

Among participants for whom both a QuICCDig and a SUS score could be calculated, a statistically significant moderate positive correlation was identified (n=54; ρ_52_=0.388; *P*=.004; 95% CI 0.120-0.604) between a user’s calculated SUS score and QuICCDig score (Figure 2). There was no statistically significant difference in the number of study completions between mock-up interfaces (n=93; *F*_8,84_=1.122; *P*=.36), amount of information presented (n=93; *F*_2,90_=1.719; *P*=.19), or consent interface type used (n=93; *F*_2,90_=0.271; *P*=.76). The calculated QuICCDig scores ranged from –0.775 to 0.775 with a mean of 0.371 (SD 0.305); the SUS scores ranged from 13 to 40 with a mean of 32.11 (SD 6.864).

**Figure 3:**
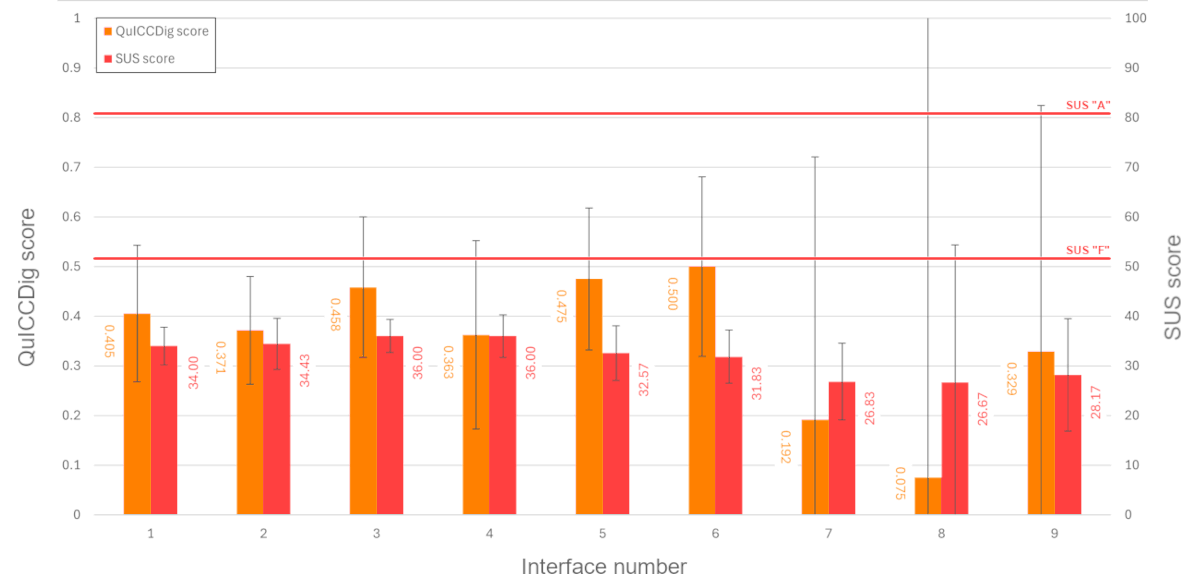
Mean Quality of Informed Consent Collected Digitally (QuICCDig) and System Usability Scale (SUS) scores by interface; 95% CIs for both are displayed in error bars. Grade boundaries for the SUS score are taken from the work of Sauro and Lewis [40].

While there were substantial numerical differences in the mean scores between interfaces, as shown in Figure 3, an ANOVA across the different mock-up interfaces for average QuICCDig score (N=55; *F*_8,46_=0.946; *P*=.49) and average SUS score (n=54, *F*_8,45_=1.629; *P*=.14) found that these differences were not statistically significant, although the averages did indicate some superiority of the first 2 information levels compared to all information being contained on the same page (Tables 4 and 5). Likewise, no significant difference was found in either SUS or QuICCDig scores based on previous e-consultation use (SUS: n=54; Mann-Whitney *U*=228.0; *P*=.44; QuICCDig: N=55; Mann-Whitney *U*=248.0; *P*=.62). Time elapsed interacting with the interfaces is shown in Table 6 but was not statistically significantly different between experiments (N=55; *F*_8,46_=0.895; *P*=.53).

Across the board, only 13% (7/55) of the participants recalled being offered alternatives to e-consultation, something presented only in the policy text itself. No correlation was observed between the number of participants who recalled being offered alternatives and the mock-up interface shown. Of the 7 participants who did remember alternatives and stated which ones they remembered, 6 correctly recalled at least 1 alternative option presented by the privacy notice, although only 1 recalled both alternatives (NHS 111 or speaking to the GP by another means), and 1 also produced an alternative which was not included in the notice (NHS 999).

A total of 55% (30/55) of the participants recalled making a privacy-related decision during their e-consultation. Of the 26 participants who responded when asked what they recalled deciding, 12 (46%) referenced the idea of their data being processed; 9 (35%) wrote that they had to agree to privacy information, while 6 (23%) recalled deciding not to read the privacy information. Only 5 (19%) participants gave a specific decision they had to make, for example, “agree [sic] to google analytics,” or “if I should use e-consultation at all […] as I was worried about data breaches.”

When asked about the impact that the use of e-consultation would have on the confidentiality of their medical records, of the 37 respondents who wrote a response, 3 (8%) gave a response identifying a specific change (“It’ll be visible to my medical provider”; “Only the transport risk”; and “The information […] may be accessible to the software company and Google”); 15 (41%) stated they did not believe there to be any change. Some participants (n=8, 22%) also expressed feeling that their data would be generically “more” or “less” secure, while 11 (30%) stated they did not know.

Notably, 29% (2/7) of the participants from interface 2 (the drag and drop group was presented with a simple privacy policy link) wrote in free text that they would have liked to have what they described as a “simplified” or “short” version of the legal information before providing consent. No participants from any of the other groups made such a comment.

Most of the participants who responded when asked in free text whether their understanding of what an e-consultation was had changed after completing the consultation stated that their understanding did not change (13/23, 57%), while a plurality stated that they thought the mock-up interface was too simple for a full e-consultation (10/23, 43%).

**Table 4:** Mean System Usability Scale scores per experiment.

	Blanket with privacy policy, mean (SD)	Summary and details page, mean (SD)	All policy information contained on a single page, mean (SD)
Checkbox	Interface 1 34.00 (5.249)	Interface 4 *36.00 (1.732)* ^a^	Interface 7 26.84 (7.360)
Drag and drop	Interface 2 34.43 (5.563)	Interface 5 32.57 (5.940)	Interface 8 26.67 (11.150)^b^
Swipe	Interface 3 *36.00 (3.162)*	Interface 6 31.84 (5.115)	Interface 9 28.17 (10.815)

^a^Italics indicate the best result.

^b^The worst result.

**Table 5:** Mean Quality of Informed Consent Collected Digitally scores per experiment.

	Blanket with privacy policy, mean (SD)	Summary and details page, mean (SD)	All policy information contained on a single page, mean (SD)
Checkbox	Interface 1 0.405 (0.192)	Interface 4 0.363 (0.063)	Interface 7 0.192 (0.504)
Drag and drop	Interface 2 0.371 (0.118)	Interface 5 0.475 (0.155)	Interface 8 0.075 (0.728)^a^
Swipe	Interface 3 0.458 (0.135)	Interface 6 *0.5 (0.172)* ^b^	Interface 9 0.329 (0.472)

^a^The worst result.

^b^Italics indicate the best result.

**Table 6:** Median interaction duration for each mock-up interface.

Interface number	Interaction duration (s), median (IQR)
1	53.3 (35.9-72.4)
2	61.0 (52.7-64.4)
3	61.0 (59.2-62.8)
4	57.9 (32.0-77.2)
5	84.0 (56.1-89.7)
6	69.3 (40.8-79.8)
7	52.0 (39.2-71.1)
8	95.3 (67.7-103.9)
9	86.0 (53.2-97.2)

## Discussion

### Principal Results

The link found between the quality of consent and perceived interface usability is a significant novel result and strongly supports the importance of high-quality interface design when building remote-access consent collection systems, such as those used for e-consultations. Designers of these systems should consider using user testing with the SUS, QuICCDig, and/or similar scoring systems to evaluate the effectiveness of their designs for gathering informed consent from users. Likewise, organizations responsible for their implementation should consider high-quality user interface design to be a requirement of such systems to effectively gather consent from patients and not merely a “nice-to-have.”

Nonetheless, across all user interfaces tested, both SUS and QuICCDig scores were objectively low. The maximum average SUS score attained by any of the interfaces was 36 for interfaces 3 and 4 (SDs 3.162 and 1.732, respectively), which is between the fourth and sixth percentile of raw SUS scores and would equate to a letter grade of F [40]. Likewise, the maximum theoretical QuICCDig score is 1, yet the highest attained score by any of the interfaces presented was 0.5. A total of 17% (9/54) of the participants who completed the full study admitted in free text, choosing not to read privacy information thoroughly or at all, even in the context of a study in which participants were informed beforehand of the aim to assess the informedness of consent, which may have had an impact on the SUS scores obtained. An interface required to complete a task that is a prerequisite but ancillary to the user’s intended goal in the consultation may therefore always receive low scores. Equally, participants’ broader attitudes to privacy and medical consent may have impacted perceived usability or desire to interact with the interface at all. The low number of participants conveying understanding of either positive or negative changes in the confidentiality of their medical records may have arisen for similar reasons. Alternative novel designs may warrant exploration, such as those incorporating voice-over or slideshow interfaces, to examine whether different modalities can deliver improvements.

Fewer than 1 in 7 participants remembered being offered alternatives to completing an e-consultation, even when every design included them in the presented information. This lack of awareness may be particularly concerning for patients less confident in completing their health interactions online, such as older patients, or for patients with what they perceive to be more serious conditions, who may feel less comfortable with online contact with their health care provider [41]. Further research should be conducted targeting these groups specifically. Real-world patients may be aware of existing alternatives anyway, such as out-of-hours services or calling their medical practice by telephone [42]. However, some patients feel pushed into e-consultation use by their primary care provider to avoid alternative methodologies such as face-to-face appointments, which may affect their trust in the service [43]. One study conducted in Norway in 2025 found that 9.5% of the patients who used a primary care e-consultation platform said that sending an e-consultation was not their first choice [42]; clear display of alternative options as part of the process of beginning a consultation may benefit these patients. Similarly, while some patients who are not English speakers may feel more confident completing an e-consultation than a telephone or face-to-face consultation [44], others may prefer to be seen in person [45]; challenges among this patient group with understanding how to contact primary care providers have been specifically highlighted in focus groups conducted by the NHS [46], underscoring the importance of this information being properly conveyed.

Both the drag-and-drop summary and swipe summary interfaces had higher average QuICCDig scores, but lower average SUS scores, than all other interfaces except for the ones containing the entire policy on the consent page. This could potentially indicate a priming effect: perhaps seeing a small amount of information begets interest in more, owing to an increased level of concern, akin to the bulletproof glass effect identified by Brough et al [47]. However, this study’s low sample size makes drawing any strong inference from this difficult; future work could investigate this effect in greater detail, perhaps through a more qualitative analysis.

### Comparison to Prior Work

Easier-to-use user interfaces appear to be associated with increased quality of consent in this study. This builds on the findings of Lindegren et al [29], demonstrating the effect of consent design patterns on usability and user attention. Habib et al [35] further found that different user interfaces could result in substantially different patterns in recall of consent information, although they did not test the usability of the interfaces they examined.

Research has previously highlighted issues surrounding a lack of reporting on the safety and quality of electronic health care consent collection. Ramos [48], writing in the context of an HIV clinic setting, pointed out that although guidelines clearly assess the accessibility of medicolegal consent information provided in paper form, no standard has existed for evaluating patients’ consent following interactions with a digital consent collection user interface. This was once again raised as an important gap in a 2024 systematic review by Leighton et al [4], noting poor reporting on safety— underscoring the fact that in nearly a decade, there has been little progress in this field.

In research medicine, many studies have evaluated the use of technological consent acquisition user interfaces. By virtue of the fact that the end users of these systems are research volunteers, they are likely to have more time and inclination to read consent information than members of the general public accessing health care services, yet even in this group, few definitive findings relating to usability exist [49]. However, usability as measured by SUS scores in research participants appears to be far better than that found in this study [50]. It is possible that participants view providing consent as the primary task in a research consent collection platform, whereas they view it as an ancillary one in a patient–primary care provider consultation interaction, and that this difference is reflected in the reduced SUS scores in this study. This would mirror the findings of Utz et al [51] that some users provide consent for cookies to be placed by websites simply as the easiest path to completing the objective of their visit, obviously with concerning implications for the quality and validity of medical consent.

This study focuses on traditional user interfaces and has not addressed the potential of conversational user interfaces (CUIs) to impact consent. Previous research has shown that users may accept or even prefer information given in this form [52]. CUIs can evoke feelings of social presence [53], and while this has been shown to feel intrusive in the e-commerce context, personal connection to clinicians during consent conversations has been shown to reduce anxiety and improve clinical outcomes [54]. Some research suggests that “chatbot” interfaces that appear overtly as a bot may produce equal perceptions of expertise as those that present themselves as a physician [55]. However, unless a bank of preset responses is used, presenting privacy information this way risks eventual “inevitable” errors [56], potentiating invalid consent based on invalid information. Further research is required in this area to evaluate the safety and efficacy of modern CUI systems in privacy-related decision-making interfaces.

### Limitations

The interpretation of these results should be tempered by the nature and size of the sample and the snowball method of acquisition. While the positive findings are still significant, the negative results may have limited generalizability, owing to the underpowering of the study—the lower-than-targeted sample size may, for instance, have prevented the detection of actual differences in the SUS or QuICCDig scores between the interface types. The sample achieved is also heavily biased toward young, well-educated participants, including those with health care– or computer science–related education. Well-educated participants appear to consistently have higher levels of comprehension and better quality of consent as a consequence [57,58]; education may also have a more direct influence on privacy-protective behavior [59]. Results have varied internationally with respect to the effect that age has on measures of consent quality and recall, although older patients, underrepresented in this study, are known to have higher rates of neurocognitive impairment than younger patients, which presents unique issues for ensuring informed consent is collected effectively wherever possible [60]. Furthermore, some evidence suggests older people may have greater privacy concerns on average than younger people [61].

The variability of health systems’ uptake of asynchronous e-consultation systems internationally and the apparent heterogeneity of different populations’ digital health literacy [62] may also limit this study’s relevance outside of the UK NHS context. The effect of using a simulated clinical interaction in which participants were aware that consent and privacy were being studied, as opposed to using real-world patients, is also unknown and limits the direct applicability of this study to clinical practice. With rigorous ethical safeguards in place, future trials conducted directly in primary care settings with larger, more representative populations should consider using real patient interactions to evaluate these systems and explore in more detail the potential for an intention-behavior gap in these interactions with respect to informed consent.

The QuICCDig scoring system has not been formally externally reviewed, and this is not a formal validation study. There is currently no clear gold-standard metric in the field to validate against, which makes designing a robust validation study challenging. Future research could consider validating against clinician-perceived informedness, although it is important to be mindful of the biases this could introduce, or perhaps simultaneously against validated scores for digital consent, clinical consent, and/or health care research consent. However, this may be difficult to orchestrate in practice.

### Conclusions

We find that contemporary user interfaces for collecting informed consent in remote electronic consultations for primary care may not reliably collect fully informed consent from patients. Furthermore, we find the perceived usability of a user interface to be significantly (*P*=.004) correlated with the quality of consent acquired through that interface. However, even in a study where participants knew consent was being studied, we find that almost half (25/55, 45%) of the participants did not remember making a privacy decision, and more than 87% (48/55) of the participants did not remember being offered alternatives to agreeing to complete their consultation. We recommend that primary care decision-makers consider the quality and usability of a remote consultation and triage application’s consent interface when making procurement decisions.

## Appendix

Multimedia Appendix 1 Screenshots of the 9 user interfaces users were presented with, demonstrating the levels of privacy information presented and the methodologies used for the consent process in each.

Multimedia Appendix 2 Quality of Informed Consent Collected Digitally questionnaire and calculation process.

## Abbreviations

CUI: conversational user interface
GP: general practitioner
NHS: National Health Service
QuICCDig: Quality of Informed Consent Collected Digitally
SUS: System Usability Scale

## Data Availability

The datasets generated or analyzed during this study are available from the corresponding author on reasonable request.
