# Correction: Quality of Informed Consent and Interface Usability in Primary Care e-Consultation: Cross-Sectional Study

**DOI:** 10.2196/103250

**Published:** 2026-08-12

**Authors:** Caitlin Parfitt-Ford, Lisa Ballard, Adriane Chapman

**Affiliations:** 1 School of Electronics and Computer Science Faculty of Engineering and Physical Sciences University of Southampton Southampton United Kingdom; 2 Primary Care, Population Science, and Medical Education Faculty of Medicine University of Southampton Southampton, England United Kingdom; 3 Southampton Biomedical Research Centre National Institute for Health and Care Research Southampton, England United Kingdom

In “Quality of Informed Consent and Interface Usability in Primary Care e-Consultation: Cross-Sectional Study” [1], the authors made one clarification.

The name of the first author was changed to Caitlin.

The correction will appear in the online version of the paper on the JMIR Publications website, together with the publication of this correction notice. Because this was made after submission to PubMed, PubMed Central, and other full-text repositories, the corrected article has also been resubmitted to those repositories.
