# Supplementary material for: Quality of Informed Consent and Interface Usability in Primary Care e-Consultation: Cross-Sectional Study
Source: JMIR Hum Factors. 2026 Feb 9;13:e78483. doi: 10.2196/78483 (PMC12930146; doi:10.2196/78483)
Supplement: Multimedia Appendix 1 [file humanfactors_v13i1e78483_app1.pdf]

Interface 1: Checkbox consent mechanism, blanket statement with link to privacy policy as information presented

Before you continue...

We'll use your personal data to make this service work. We also use cookies to help us understand the performance and usage of our site.

Read our privacy policy

Agree to continue

Interface 2: Drag-drop consent mechanism, blanket statement with link to privacy policy as information presented

Before you continue...

We'll use your personal data to make this service work. We also use cookies to help us understand the performance and usage of our site.

Read our privacy policy

Drag me to consent

Drop here to confirm consent and continue

Interface 3: Swipe consent mechanism, blanket statement with link to privacy policy as information presented

Before you continue...

We'll use your personal data to make this service work. We also use cookies to help us understand the performance and usage of our site.

Read our privacy policy

Slide to agree

Interface 4: Checkbox consent mechanism, summary of privacy policy and details page as information presented

Before you continue...

We work with your GP to process your healthcare data for them - the only people with access to the data you put in here are people at your GP surgery, and our own team to provide the service.

We also use Google Analytics to anonymously understand how our site is used.

If you'd like to understand more, you can:

Read our privacy policy

☐ Agree to continue

Interface 5: Drag-drop consent mechanism, summary of privacy policy and details page as information presented

Before you continue...

We work with your GP to process your healthcare data for them - the only people with access to the data you put in here are people at your GP surgery, and our own team to provide the service.

We also use Google Analytics to anonymously understand how our site is used.

If you'd like to understand more, you can:

Read our privacy policy

Drag me to consent

Drop here to confirm consent and continue

Interface 6: Swipe consent mechanism, summary of privacy policy and details page as information presented

Before you continue...

We work with your GP to process your healthcare data for them - the only people with access to the data you put in here are people at your GP surgery, and our own team to provide the service.

We also use Google Analytics to anonymously understand how our site is used.

If you'd like to understand more, you can:

Read our privacy policy

Slide to agree

Interface 7: Checkbox consent mechanism, all policy information contained on a single page as information presented

Before you continue...

This privacy policy sets out how we collect and use the personal data you provide when using our electronic medical consultation application. Please read this privacy policy carefully to understand how we use your personal data.

We are a data processor for your GP. This means that we process personal data on behalf of your GP to provide the electronic medical consultation service. Your GP is the data controller for the personal data we collect from you, and is responsible for ensuring that the personal data we process on their behalf is used in accordance with data protection laws. You should refer to your GP's privacy policy for more information on how they use your personal data.

We use analytics providers such as Google Analytics to understand how our site is being used. This helps us improve the service we offer and make it more user-friendly. The personal data we collect from you will not be used for any other purposes, and will only be used to provide the electronic medical consultation service.

When you use our electronic medical consultation application, we will collect the personal data you provide, such as your name, date of birth, and symptoms. This personal data is used only for healthcare purposes and for providing the service. Once the consultation is finished, all the data collected will be sent immediately to your GP.

We will only access the personal data you provide for technical reasons to facilitate providing the service. We will retain the personal data for not longer than one month. We will not share your personal data with anyone other than your GP and their subprocessors.

Please note that special category data relating to healthcare may be collected when you use our electronic medical consultation application. We have appropriate safeguards in place to protect this data.

All personal data we collect from you will be kept within the UK and will not be shared with any other countries.

We will do everything we can to prevent security breaches from happening, but we cannot guarantee that they will not happen. We also cannot guarantee the security of your own computer. If you are not happy to agree to this privacy policy, you can call your GP surgery or NHS 111 for alternative services. If you have any questions about how your personal data is used and what happens during an e-Consultation, or if you would like your data to be deleted, you can call your GP to discuss this.

☐ Agree to continue

Interface 8: Drag-drop consent mechanism, all policy information contained on a single page as information presented

Before you continue...

This privacy policy sets out how we collect and use the personal data you provide when using our electronic medical consultation application. Please read this privacy policy carefully to understand how we use your personal data.

We are a data processor for your GP. This means that we process personal data on behalf of your GP to provide the electronic medical consultation service. Your GP is the data controller for the personal data we collect from you, and is responsible for ensuring that the personal data we process on their behalf is used in accordance with data protection laws. You should refer to your GP's privacy policy for more information on how they use your personal data.

We use analytics providers such as Google Analytics to understand how our site is being used. This helps us improve the service we offer and make it more user-friendly. The personal data we collect from you will not be used for any other purposes, and will only be used to provide the electronic medical consultation service.

When you use our electronic medical consultation application, we will collect the personal data you provide, such as your name, date of birth, and symptoms. This personal data is used only for healthcare purposes and for providing the service. Once the consultation is finished, all the data collected will be sent immediately to your GP.

We will only access the personal data you provide for technical reasons to facilitate providing the service. We will retain the personal data for not longer than one month. We will not share your personal data with anyone other than your GP and their subprocessors.

Please note that special category data relating to healthcare may be collected when you use our electronic medical consultation application. We have appropriate safeguards in place to protect this data.

All personal data we collect from you will be kept within the UK and will not be shared with any other countries.

We will do everything we can to prevent security breaches from happening, but we cannot guarantee that they will not happen. We also cannot guarantee the security of your own computer. If you are not happy to agree to this privacy policy, you can call your GP surgery or NHS 111 for alternative services. If you have any questions about how your personal data is used and what happens during an e-Consultation, or if you would like your data to be deleted, you can call your GP to discuss this.

Drag me to consent

Drop here to confirm consent and continue

Interface 9: Swipe consent mechanism, all policy information contained on a single page as information presented

Before you continue...

This privacy policy sets out how we collect and use the personal data you provide when using our electronic medical consultation application. Please read this privacy policy carefully to understand how we use your personal data.

We are a data processor for your GP. This means that we process personal data on behalf of your GP to provide the electronic medical consultation service. Your GP is the data controller for the personal data we collect from you, and is responsible for ensuring that the personal data we process on their behalf is used in accordance with data protection laws. You should refer to your GP's privacy policy for more information on how they use your personal data.

We use analytics providers such as Google Analytics to understand how our site is being used. This helps us improve the service we offer and make it more user-friendly. The personal data we collect from you will not be used for any other purposes, and will only be used to provide the electronic medical consultation service.

When you use our electronic medical consultation application, we will collect the personal data you provide, such as your name, date of birth, and symptoms. This personal data is used only for healthcare purposes and for providing the service. Once the consultation is finished, all the data collected will be sent immediately to your GP.

We will only access the personal data you provide for technical reasons to facilitate providing the service. We will retain the personal data for not longer than one month. We will not share your personal data with anyone other than your GP and their subprocessors.

Please note that special category data relating to healthcare may be collected when you use our electronic medical consultation application. We have appropriate safeguards in place to protect this data.

All personal data we collect from you will be kept within the UK and will not be shared with any other countries.

We will do everything we can to prevent security breaches from happening, but we cannot guarantee that they will not happen. We also cannot guarantee the security of your own computer. If you are not happy to agree to this privacy policy, you can call your GP surgery or NHS 111 for alternative services. If you have any questions about how your personal data is used and what happens during an e-Consultation, or if you would like your data to be deleted, you can call your GP to discuss this.

Slide to agree
