# Supplementary material for: Quality of Informed Consent and Interface Usability in Primary Care e-Consultation: Cross-Sectional Study
Source: JMIR Hum Factors. 2026 Feb 9;13:e78483. doi: 10.2196/78483 (PMC12930146; doi:10.2196/78483)
Supplement: Multimedia Appendix 2 [file humanfactors_v13i1e78483_app2.pdf]

QuICCDig calculation

Question	Method	Scoring	Max score	Equal weighted score
Were you able to successfully complete your e-consultation?	Yes/no	-10 points for no; 1 points for yes	1	0.1
How easy did you find it to complete your e-consultation?	Five-level Likert from “Very easy” (4 points) to “Very difficult” (0 points)	Likert scoring	4	0.025
Did you get an explanation of what an e-consultation involves?	Yes/no	1 point for yes	1	0.1
How well do you feel you understand e-consultations now?	Five-level Likert: “A lot” (4 points), “I get most of it” (3), “I get the basics” (2), “A little bit” (1), “I don’t understand at all” (0 points)	Likert scoring	4	0.025
How long will your consultation data be stored?	Select one: Only until they’re read by a healthcare provider, Up to a month, Up to six months, Up to a year, Up to ten years, Forever	1 point for correct answer, 0 for incorrect	1	0.1
Which of the below might have access to some data collected during the consultation?	Select multiple: Me, my healthcare provider, other healthcare providers, the company that runs the consultation software,	1 point for each correct answer	4	0.025

	Google, Facebook			
Which of the below risks involved in the e-consultation process were you told about?	Select multiple: The healthcare provider might lose my consultation and I have to submit it again, The software company might have a security breach and my data might be compromised, There might be an attacker on my network that steals my data, My computer might have malware that can access my submitted information, The computer might accidentally send my consultation to another healthcare provider, My healthcare provider might not be able to fully assess me only on the basis of the information I provide	1 point for each correct answer, -1 point for incorrect answers	4	0.025
Do you remember being told about the alternatives to using an e-consultation?	Yes/no	1 point for yes	1	0.1
How satisfied with the decision-making process for whether you wanted to continue with an e-consultation were you?	Standard 5-level Likert	Likert scoring	4	0.025
To what extent did	Five-level Likert: ”Not at	Likert	1	0.1

you feel informed and involved with the process of consenting for your e-consultation?	all" (-2), "Somewhat" (-1), "Enough" (1), "More than enough" (0), "Too much" (-1)	scoring
